# Enhanced Spine Stability and Survival Lead to Increases in Dendritic Spine Density as an Early Response to Local Alpha-Synuclein Overexpression in Mouse Prefrontal Cortex

**DOI:** 10.1007/s10571-024-01472-7

**Published:** 2024-04-26

**Authors:** Peter J. Bosch, Gemma Kerr, Rachel Cole, Charles A. Warwick, Linder H. Wendt, Akash Pradeep, Emma Bagnall, Georgina M. Aldridge

**Affiliations:** 1 Department of Neurology, Carver College of Medicine, University of Iowa, 169 Newton Road, Pappajohn Biomedical Discovery Building, Iowa City, 52242 USA; 2 Department of Pharmacology, University of Iowa, Iowa City, USA; 3 Iowa Neuroscience Institute, University of Iowa, Iowa City, IA USA; 4 Institute for Clinical and Translational Science, University of Iowa, Iowa City, IA USA

**Keywords:** Alpha-synuclein, Dendrite, Dendritic spine, Parkinson’s disease, Lewy body dementia, Neuron, Overexpression, Prefrontal cortex, 2-photon, Synucleinopathy, Thy1-YFP-H, Yellow fluorescent protein, YFP, Phosphorylated alpha-synuclein

## Abstract

Lewy Body Dementias (LBD), including Parkinson’s disease dementia and Dementia with Lewy Bodies, are characterized by widespread accumulation of intracellular alpha-Synuclein protein deposits in regions beyond the brainstem, including in the cortex. However, the impact of local pathology in the cortex is unknown. To investigate this, we employed viral overexpression of human alpha-Synuclein protein targeting the mouse prefrontal cortex (PFC). We then used in vivo 2-photon microscopy to image awake head-fixed mice via an implanted chronic cranial window to assess the early consequences of alpha-Synuclein overexpression in the weeks following overexpression. We imaged apical tufts of Layer V pyramidal neurons in the PFC of Thy1-YFP transgenic mice at 1-week intervals from 1 to 2 weeks before and 9 weeks following viral overexpression, allowing analysis of dynamic changes in dendritic spines. We found an increase in the relative dendritic spine density following local overexpression of alpha-Synuclein, beginning at 5 weeks post-injection, and persisting for the remainder of the study. We found that alpha-Synuclein overexpression led to an increased percentage and longevity of newly-persistent spines, without significant changes in the total density of newly formed or eliminated spines. A follow-up study utilizing confocal microscopy revealed that the increased spine density is found in cortical cells within the alpha-Synuclein injection site, but negative for alpha-Synuclein phosphorylation at Serine-129, highlighting the potential for effects of dose and local circuits on spine survival. These findings have important implications for the physiological role and early pathological stages of alpha-Synuclein in the cortex.

**Graphical Abstract:**

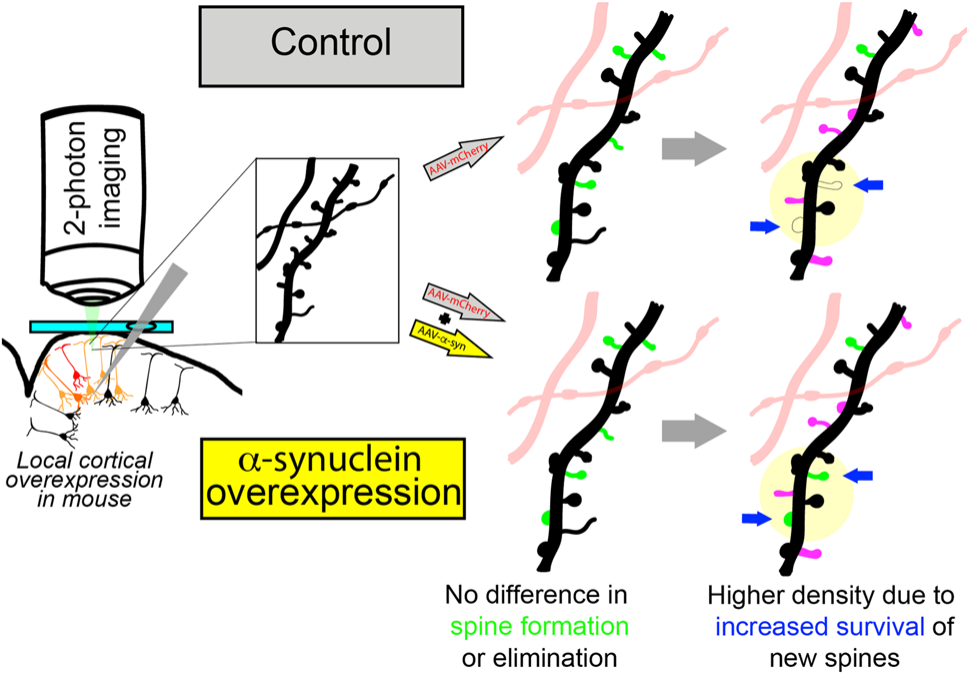

**Supplementary Information:**

The online version contains supplementary material available at 10.1007/s10571-024-01472-7.

## Introduction

Lewy Body Dementias (LBD), which include both Parkinson’s disease dementia (PDD) and Dementia with Lewy bodies (DLB) are debilitating multi-system diseases that result in unpredictable hallucinations, fluctuations in cognition, disturbed sleep, delusions, and depression (Weintraub et al. 2022). Cognitive impairment in PDD/DLB differs dramatically from patients with other forms of dementia; it is characterized by deterioration of executive functions, such as cognitive control, timing, attention, planning, and cognitive flexibility (Aldridge et al. 2018; Smirnov et al. 2020). As prefrontal cortex (PFC) circuits modulate executive functions, pathology in this region may play a major role in some cognitive symptoms of LBD (Gnanalingham et al. 1997; Walker et al. 2015; Zhang et al. 2021).

Lewy Bodies are large intracellular aggregates that contain the highly expressed protein, alpha-Synuclein (α-Syn). Pathologically, PD is characterized by the presence of “Lewy Bodies” in the midbrain, in combination with pallor indicative of loss of dopaminergic neurons in that location (Obeso et al. 2017). By contrast, DLB and PDD cases are often distinguished from PD cases pathologically by an increased number of diffuse cortical or limbic aggregates also containing α-Syn, but which can differ in morphology from brainstem Lewy Bodies (McKeith et al. 2017). In both LBD and PD, the majority of α-Syn in aggregates is phosphorylated at Serine-129 (pSer129) (Anderson et al. 2006; Fujiwara et al. 2002; Colom-Cadena et al. 2017). Duplication or triplication of the α-Syn gene, SNCA, leads to familial forms of autosomal-dominant early onset PD and LBD (Spellman 1962; Singleton et al. 2003). Consistent with the hypothesis that the magnitude of α-Syn expression contributes to disease onset, triplication of SNCA leads to earlier onset and more rapid progression than duplication (Chartier-Harlin et al. 2004). However, some individuals with duplication have been reported to have psychiatric manifestations even in childhood (Konno et al. 2016; Elia et al. 2013). Efforts to tease apart the role of α-Syn overexpression in animal models have mostly focused on deterioration of motor function. It is noteworthy that patients with SNCA triplication are at higher risk for cognitive dysfunction (Chartier-Harlin et al. 2004; Fuchs et al. 2007), whereas some mutations in SNCA lead to parkinsonism with less cognitive effect (Planas-Ballvé and Vilas 2021).

Although cell loss (including dopaminergic and cholinergic projection neurons) undoubtably contributes to some of the symptoms characteristic of LBD, there are examples of individuals with pathology in the cortex with less severe pathology in the brainstem (Zaccai et al. 2008). Associative cortices, including PFC, often show α-Syn pathology in patients with DLB and PDD (Harding and Halliday 2001), and Lewy bodies in the frontal cortex were found in one study to be the strongest predictor of cognitive impairment (Mattila et al. 2000). There is evidence from a small number of LBD autopsies that the PFC has decreased density of dendritic spines in the setting of micro-aggregates of α-Syn at the pre-synapses in this region (Kramer and Schulz-Schaeffer 2007). Although the prior study did not account for co-pathology, a more recent study suggests phosphorylated α-Syn correlated better than other pathologies (amyloid plaques or tau tangles) with loss of synaptic markers (Frigerio et al. 2024). However, not all studies find evidence for synapse loss in pure LBD (Hansen et al. 1998). How local α-Syn impacts cortical neurons and circuits remains a critical unanswered question.

Prior studies have tried to address these questions with animal models. Transgenic overexpression of human α-Syn in mouse (Blumenstock et al. 2017) and α-Syn preformed fibril injections into dorsal striatum after 5 months were associated with decreases in spine density in Layers I and Layer IV/V cortical neurons (Blumenstock et al. 2017). Alterations in dendritic spines in the absence of cell death could have important implications; dendritic spines are the post-synaptic component of most excitatory synapses, and spines can exhibit plasticity in response to experience (Hedrick et al. 2022; Nava et al. 2017; Pan et al. 2010). However, most prior studies examining the relationship of α-Syn to synaptic changes did not limit pathology to the cortex, and thus such findings may have been driven by changes in neurotransmitter levels.

In our previous work, we found an increased dendritic spine density in the medial PFC following local α-Syn overexpression, using a single post-mortem timepoint paired with Golgi staining (Wagner et al. 2020). Our data suggested that 10 weeks following injection, mice with α-Syn overexpression had greater local spine density than mice injected with a control virus. Here, we used longitudinal 2-photon transcranial imaging to test the specific hypothesis that changes in spine density were due to increased spine formation. We examined Layer V neuronal tufts (visualized in Layer I) on a weekly basis, evaluating the same dendrites before and after local viral overexpression of human α-Syn. We found that α-Syn overexpression initially increases local dendritic spine density beginning 5-weeks post-AAV injection. Furthermore, in contrast to our original hypothesis, the increase appears to be secondary to increased new-spine stability and survival in the region of overexpression. These findings may have implications both for Lewy body diseases, as well as other conditions, such as development, infection, and autism, where alterations in expression of this abundant presynaptic protein are being investigated (Karaca et al. 2023; Kasen et al. 2022).

## Methods

### Animals and Treatments

All experiments involving mice were approved by the University of Iowa Institutional Animal Care and Use Committee (Protocol #9082242 and 2122242). All experiments were performed in strict adherence with the National Institute of Health (NIH) Guide for the Care and Use of Laboratory Animals. Mice were handled until comfortable with experimenter handling and with the Neurotar setup before any imaging sessions commenced.

For in vivo spine analysis studies, we utilized the Thy1-YFP mouse line, encoding the fluorescent molecule, YFP under the control of the Thy1 promoter: B6.Cg-Tg(Thy1-YFP)HJrs/J; stock number 3782 (Jackson Labs), and the Thy1-GFP line for confocal imaging studies: Tg(Thy1-GFP)MJrs/J; stock number 007788 (Jackson Labs). For Thy1-YFP, males and females were used and randomized to either control (*n* = 6) or α-Synuclein overexpression (*n* = 8) treatment groups prior to experiment onset. All mice were injected as mature adults, with age range for controls 39–70 weeks old; α-Synuclein overexpression range 40–73 weeks old. The Thy1-GFP mice contained 4 mice (3 m, 1F); age at injection ranged from 11 to 24 weeks old. Mice were housed in groups of 2–4 per cage, except when directed to singly house by the facility veterinarian due to fighting. In general, the cranial window and head bar did not prevent group housing.

### Cranial Window Surgery

Cranial window implantation was performed as in our prior studies (Keyes et al. 2021) with changes as noted. Cranial window surgeries in Thy1-YFP mice were performed using ketamine/xylazine for anesthesia. Under deep anesthesia, animals were administered Meloxicam (2 mg/kg, IP) and Bupivacaine (8 mg/kg, SQ) prior to shaving and cleaning of the surgical site with Betadine surgical scrub and 70% ethanol. The skull was exposed using a clean incision from anterior to posterior with surgical scissors, periosteum was removed, and a 3-mm-diameter round craniotomy was made using a dental drill with a 0.5 mm bit (Fine Science Tools, 1900705). Cranial windows were installed covering a region − 0.5 mm bregma to 2.5 mm bregma (AP), 2.5 mm on the left, and 0.5 mm on the right. The craniotomy crossed the sagittal suture and included the future injection site location. The dura was removed in all animals at the time of cranial window implant over the imaging region, to allow penetration of a pulled glass pipette with limited disruption to the imaging location. The exposed brain was covered using Kwik-Sil silicon adhesive (WPI, Cat: KWIK-SIL), and the 3 mm coverslip window was placed to cover the craniotomy before being sealed with cyanoacrylate. The coverslip contained a pre-drilled hole, which was later used to inject viral vector into the brain. Two skull screws (McMaster-Carr (90910A310), #000-120) were placed over occipital cortex, inserted only deep enough to securely hold the skull. A model 11 stainless-steel lightweight headplate (Neurotar Ltd, Finland) was attached securely to the skull using cyanoacrylate followed by Teets dental cement (Methyl methacrylate). Mice were given normal saline (0.9% NaCl, SQ) post-surgery and allowed to recover for 3 weeks prior to imaging onset.

## Brain Injections

### Thy1-YFP for 2-Photon Microscopy

**Fig. 1:**
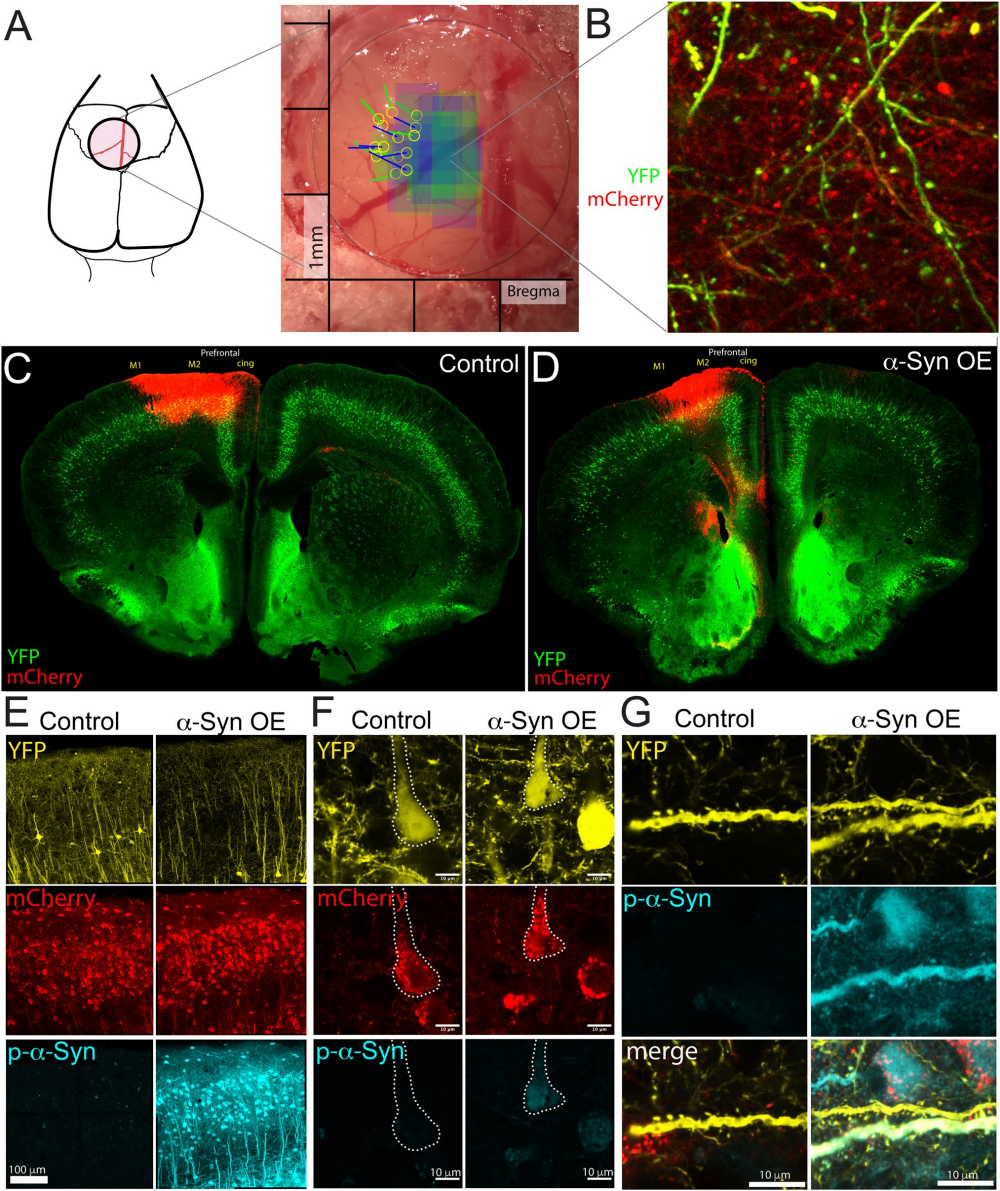
Experimental design for overexpression of human alpha-Synuclein in the mouse PFC. **A** We placed round cranial windows over the putative imaging site. AAV injections were performed after two imaging sessions, through a pre-drilled hole plugged with silicon. Injection sites for mice in the study are shown as yellow circles, with the lines (blue = control, green = α-Syn OE) representing the angle of injection. The colored boxes (blue = control, green = α-Syn OE) represent the imaging sites of apical tufts. **B** Representative live image from a highly transduced area using 2-photon. **C**–**D** Example of the localized spread of transduction in a Thy1-YFP coronal section, showing AAV-induced mCherry expression in a section from control (**C**) and α-Syn OE (**D**), localized to the mPFC (M2, cingulate) region. **E** Overexpression of human-α-Syn leads to robust phosphorylation at Ser129 in treated mice, including in soma (**F**) and some dendrites (**G**). **E**; Tiled confocal image, scale bar 100um. **F** single confocal slice, 40 × objective, scale bar 10um. **G** Max projection of 3-confocal slices, 0.33um step size

**Fig. 2:**
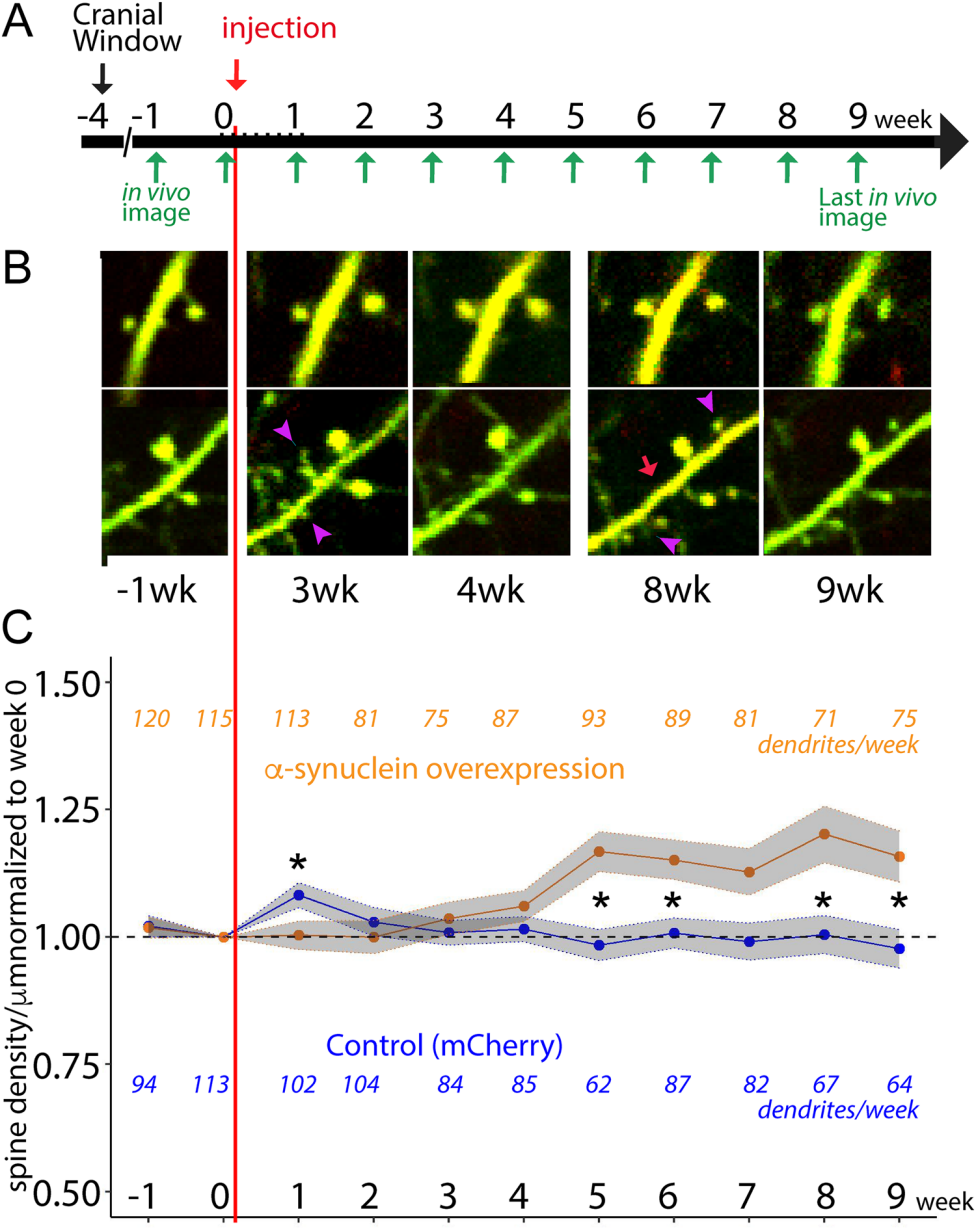
Alpha-Synuclein overexpression causes increases in dendritic spine density on individual dendrites. **A** 2-photon imaging was performed 3 weeks after cranial window surgery to generate 2 weeks of pre-treatment imaging of dendrites (week -1 and week 0, followed by injection of AAV-mCherry (control) or AAV-mCherry/α-Syn (α-Syn OE) immediately after the week 0 imaging session. Nine weeks of 2-photon microscopy was performed on the same pool of dendrites that were established in weeks − 1 and 0. **B** Representative, single plane images of control (top) and α-Syn OE (bottom) dendrites. Purple arrowheads represent new spines; red arrow is a lost spine, each identified by comparison of the full 3D stacks, not visible here. **C** Dendritic spine density increases in α-Syn OE animals compared with control. Each dendrite was divided by its pre-treatment density to evaluate the effect on individual dendrites. The orange and blue colored numbers represent the number of dendrites measured for each group at each week from *n* = 6 control, *n* = 8 α-Syn OE; dendrites were excluded at some weeks due to changes in the cranial window transparency or angle, equipment malfunction, and the covid pandemic. α-Syn OE led to increased dendritic spine density starting at week 5 and continuing for the duration of imaging (*p < 0.05)

For Thy1-YFP mice, we used the following viral treatments: Control: AAV6-CAG-mCherry-WPRE (1.7 × 10^12 gc/mL, Vector Biolabs, unilateral); α-Syn overexpression: vector carrying cDNA coding for human, wild-type α-Synuclein: AAV6-CAG-hSNCA-WPRE (1.0 × 10^13^gc/mL, VectorBiolabs) was mixed with the mCherry virus outlined above. The ratio of synuclein to mCherry in the treatment group was adjusted from 3:1 to 1:1 following discovery of interference between mCherry and YFP fluorescence (*n* = 3 synuclein mice). Animals were prepared for trans-window injection by cleaning the window and silicon plug as described for surgery. Anesthesia was induced using Isoflurane anesthesia using the Somnosuite® low flow anesthesia system (Kent Scientific). For Thy1-YFP mice, injections were done within 24 h following imaging at “week 0” (Fig. 2A). Injections were made through the pre-existing hole in the glass coverslip through a silicon seal that was located approximately 1.25 mm lateral to midline (Fig. 1A). A pulled glass pipet, pre-loaded with virus, was directed with a 20 to 40-degree angle into the brain depending on the location of the hole relative to the target imaging region. 100–200 nL of virus was injected at 5–6 locations along a track under the window, at approximately 0.4 mm, 0.8 mm, 1.2 mm, 1.6 mm, and 2 mm below the surface of the brain (maximum 1.2 µL total). Variations in amount given reflect adjustments made to better target cells in the imaged region but were matched between groups.

### Thy1-GFP for Confocal Microscopy

The Thy1-GFP mice (*n* = 4) received bilateral injections with control virus into one hemisphere and α-Synuclein overexpression into the other hemisphere: Control: AAV6-CAG-mCherry-WPRE (1.7 × 10^12^ gc/mL, Vector Biolabs, 1 μL, left hemisphere) and overexpression: vector carrying cDNA coding for human, wild-type α-Synuclein: AAV6-CAG-hSNCA-IRES-mCherry-WPRE (1.0 × 10^12^ gc/mL, VectorBiolabs, 1 μL right hemisphere). This vector has an internal ribosomal entry site (IRES) used to express mCherry in all α-Syn overexpression cells. For Thy1-GFP mice, we injected straight down (no angle). After the skull was exposed, bilateral craniotomies were drilled using a 0.5 mm drill bit at the following locations from bregma: 0.75 mm medial–lateral (ML), ± 1.4 mm anterior–posterior (AP), and − 1.0 mm DV. As with the in vivo experiment, virus (total 1.0 μL/ hemisphere) was injected using gentle air pulses through a solenoid attached to an air supply through a pulled glass pipet at an approximate rate of 100 nL/min. A greater proportion of virus (~ 600 nL) was aimed at Layer V, and the remaining (~ 400 nL) was targeted to Layer II/III along the injection track. Following the completion of injection, the needle was left in place for 5 min prior to withdrawal. After injection, the skin was sutured, normal saline (0.9% NaCl, SQ) was administered, and the mouse was allowed to recover for 6 weeks prior to trans-cardial perfusion and brain collection.

### 2-Photon Imaging

Mice were introduced to the Neurotar (Neurotar Ltd) air Table 2 weeks prior to the first imaging session, and imaging began approximately 3 weeks after the cranial window surgery. Neurotar® is a tracking system and air table setup that allows animals to run on a free-floating carbon cage while head fixed using a surgically implanted headplate. An overview dendritic map of the region was generated the week prior to the first spine imaging session. Imaging of YFP and mCherry were done using the following conditions: 935 nm laser wavelength, 3% offset, 0.5 mm step size, 320 × 320 pixels (0.1989 µm/pixel), and 8 × digital zoom. Voltage of the laser was adjusted dynamically for depth throughout the z-stack, as well as overall window clarity. The 25 × objective correction collar was set at 0.17 for water, and 0.23 for aqueous jelly (Aquasonic clear ultrasound gel, Parker Laboratories Inc, diluted 4 × in water). Backfill setting was adjusted for the highest resolution. Imaging sessions lasted up to 1 h using an upright Olympus multiphoton FVMPE-RS equipped with Mai Tai DeepSee tunable laser set at 935 nm and a 25 × water-immersion objective (N.A. = 1.05, Olympus). Mice were imaged each week for which chosen dendrites were clearly visible, up to 11 weeks consecutive weeks. In several animals, imaging was delayed beyond two weeks after window surgery to allow further clearing of the window. Prior to injection of virus, 10–20 different locations were imaged within the cranial window. Following injection of virus, we reduced the imaging locations to only those within 1 mm of the viral injection site, as determined by visualization of mCherry by 2-photon microscopy.

### Immunostaining

Six weeks after injection, Thy1-GFP animals were heavily anaesthetized using ketamine/xylazine and trans-cardial perfusion was performed using cold 4% paraformaldehyde (PFA). Brains were removed, post-fixed overnight in PFA, and cryoprotected using 30% sucrose in 1 × Phosphate-Buffered Saline (PBS, Gibco, #14190). Brains were frozen in OCT (Fischer, 4585) and cryosectioned at 100 µm thickness to maximize the dendritic arbor that could be imaged. For immunostaining procedures, sections were blocked (2% Normal Goat serum (Aldrich-Catalog: G9023, 0.3% Triton X-100 in PBS (Fisher Scientific P185111) and incubated with anti-GFP (chicken, Invitrogen, A10262, RRID: AB_2534023, 1:100) and anti-phospho-synuclein (pSer129 rabbit, Abcam, EP1536Y (AB51253), RRID: AB_869973, 1:500) primary antibodies diluted in blocking solution for 24 h at 4 °C with gentle agitation. pSer129-α-Syn antibody specificity was evaluated by comparing the region injected with AAV-α-Syn to nearby un-injected regions. Un-injected regions did not show any significant staining. Prior publications have also shown the specificity of this antibody, demonstrating no staining in α-Syn knockout mice (Delic et al. 2018). Sections were washed with PBS and incubated with the relevant secondary antibody conjugated to Alexa-Fluor 488 nm (anti-chicken, A-11039, Invitrogen, RRID AB_2534023) or 647 nm (anti-rabbit, ab150079, Abcam, RRID AB_2534023) overnight in blocking solution at 4 °C with agitation. They were then washed with PBS and mounted using Prolong™ glass Antifade Mountant (ThermoFisher, Cat: P36961) and cured for 48–60 h prior to imaging. Confocal imaging was performed using a Leica TCS Sp5 microscope and images were collected using LAS-AF software. Confocal images were taken with 0.33 µm step size, using the appropriate collection methods for the relevant Alexa-Fluor molecules used in immunostaining. Images were analyzed using Imaris (see below) and adjusted in FIJI (Schindelin et al. 2012) or Adobe photoshop only for presentation.

### Analysis

#### 2-Photon Imaging Analysis

Two photon imaging analysis was performed using FIJI. Dendrites and counting bins were selected based on predetermined criteria. Bins started at least 10 µm below the pial surface, and at least 10 µm away from any dendrite termination (potential growth cone). Bins were selected for analysis based on a target bin length of 15–25 µm and a minimum of 3 spines pre-injection. For analysis, two individuals blinded to the treatment group identified spines in all consecutive imaging weeks. Spines were counted if they were at least 0.45 µm long (> 2 pixels). Spines that unambiguously disappeared for one week, then reappeared in a similar position the following week was recorded as new spines. To monitor transduction in the region of interest, the presence of mCherry in the dendrite was recorded, as well as the number of nearby transduced neurites within the imaging location of the dendrite (~ 60 µm × 60 µm). Dendrites were defined as transduced if mCherry was visible in the dendrite when imaged using laser excitation of 1040 nm. Regions that contained local transduced neurites were scored as 0 (for no transduced neurites visible), low (for 1–5 neurites visible), or high (for 6 or greater neurites visible in the region).

For spine survival analyses, only spines that we recorded as ‘new’ during week 1–3 were included. The number of weeks were counted from the week a spine appeared until the week it was recorded lost or censored, and then survival curves were generated. Kaplan–Meier curves and analysis were performed using Prism 10 (GraphPad 10.2.0).

#### Confocal Imaging Analysis

Images that were collected on the Leica Sp5 microscope were collected as .lif files and imported directly into Imaris Microscopy Image analysis software (Oxford Instruments). Filaments were traced along basilar dendrites beginning at least 20 µm from the soma by an experimenter blinded to treatment conditions. Filaments which represented dendrites were used to automatically trace spines in Imaris, and each spine was manually checked for accuracy and corrected if required. Filaments were not traced within 5 µm of a branchpoint or 10 µm from the dendrite termination. All relevant data were exported in.csv format file, and relevant data were extracted using custom R scripts and/or excel pivot tables. GraphPad Prism was then used to analyze the data, divided into 3 groups based on the treatment hemisphere and transduction status of the parent soma: control hemisphere/mCherry-negative; α-Syn OE hemisphere/pSer129-α-Syn positive; and α-Syn OE hemisphere/pSer129-α-Syn-negative. The transduction region was defined based on the presence of mCherry (and pSer129-α-Syn staining) in the surrounding cells/dendrites, approximately 1 mm from the center of the injection. Full cell reconstruction from the confocal images was made using the FIJI plugin, “Pairwise stitching,” to stitch images together and the measure function was used in FIJI for the distance from soma to the center of the analyzed filament bin.

### Statistics

**Fig. 3:**
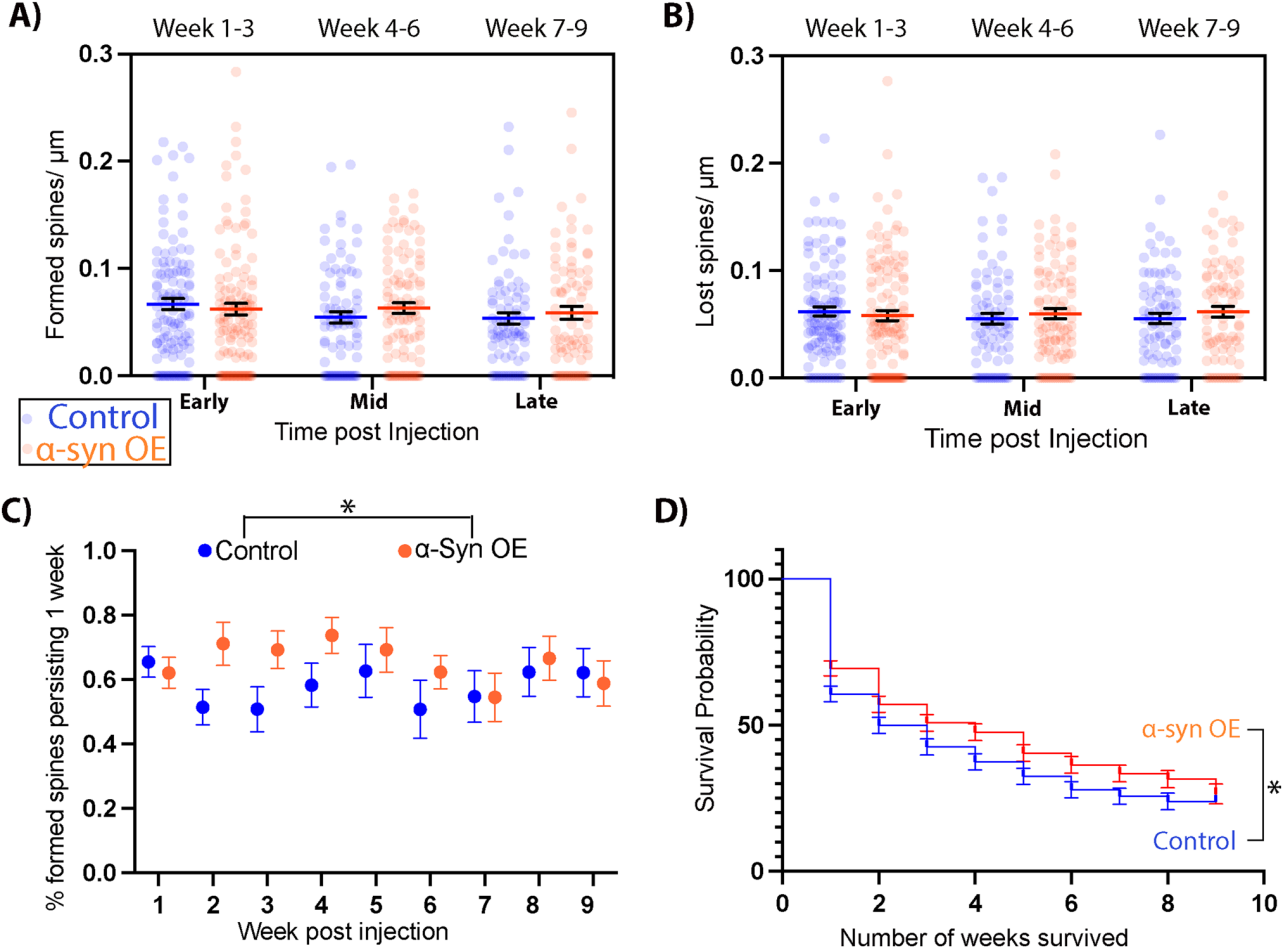
Increases in spine density following α-Syn overexpression (α-Syn OE) are due to increased spine persistence. The increase in spine density could not be attributed to significant differences in Spine Formation (**A**) or Spine Elimination (**B**). Persistence (**C**): The percent of new spines, grouped by dendrite, that persisted for at least one week following their appearance was higher in the α-Syn OE mice. Odds ratios (OR) for spine survival by TREATMENT (α-syn vs. control): OR 1.30 CI 1.01–1.67, *p* = 0.040) compared with controls, with no significant main effect of time. Survival (**D**): Spines that first appeared in week 0,1, or 2 survived significantly longer in the α-Syn OE mice compared with these spines in the control-injected mice (*p* = 0.023, Log-rank comparison of Kaplan–Meier curve. Dendrites (**A**–**C)** and spines (**D**) are from 6 control mice and 8 α-Syn OE mice

Statistics were completed using R version 4.3.1, (for density) and GraphPad Prism (for turnover, persistence, morphology and survival), and cross-checked by separate investigators. Data analysis for Fig. 2C (spine density) and Fig. 3C was performed by the Biostatistics core at the University of Iowa Institute for Clinical and Translational Science. For the analyses presented in Fig. 2C, all follow-up spine densities were divided by that dendrite’s baseline density to obtain a measure of proportional change over time. Then a linear mixed effect model was fit to assess the effect of time, treatment, and the interaction between these two variables on this proportional change in density measure. A random intercept was used for each dendrite to account for inherent between-dendrite variability. The analyses presented in Fig. 3C used a logistic mixed-effects model in which the odds of a spine being persistent were modeled with time and treatment group serving as the predictor variables. Once again, a random intercept was used for each dendrite to account for inherent between-dendrite variability. Additional details and justification for other statistical tests are provided within the results section for clarity. *P *values less than 0.05 were considered statistically significant for all analyses.

## Results

### Alpha-Synuclein Overexpression Dynamically Increases Dendritic Spine Density in Apical Tufts of Layer V Cells in the Prefrontal Cortex

Using the Thy1-YFP transgenic mouse line and AAV-mediated overexpression of human α-Syn in the PFC, we studied the dynamic changes in dendritic spines in awake, head-fixed mice using 2-photon microscopy. Thy1-YFP mice (Feng et al. 2000) express yellow fluorescent protein (YFP) in a subset of Layer V (and a smaller number of Layer II/III) neurons in the cerebral cortex, which fill the whole cell with YFP, including neurites and dendritic spines. We, therefore, used this mouse line to perform repeated imaging of dendrites and their dendritic spine protrusions. Our strategy combined cranial windows with intracranial injections through a pre-drilled hole, which had been plugged with silicon to allow for injection needle entry (Fig. 1A, B). Both groups received unilateral injections of AAV. Control animals received AAV-mCherry. α-Syn overexpression (α-Syn OE) mice received a mixed combination of the AAV-α-Syn and AAV-mCherry. We targeted the PFC, mainly encompassing the M2 (Secondary motor), Cg1 (Cingulate), and PrL (Pre-limbic) regions; some overexpression also extended into the M1 region (Fig. 1C, D). Immunofluorescence for pSer129-α-Syn and confocal microscopy demonstrated robustly expressed pSer129-α-Syn throughout transduced cells, with strong expression in the soma and nuclei of neurons. Many dendrites and spines also displayed positive pSer129-α-Syn staining at 70 days post-injection (Fig. 1E–G).

Mice were implanted with cranial windows and a secure headplate, which stabilized the head while the mouse could run on a floating platform during imaging to minimize stress. This strategy provided stable imaging and sufficient consistency to image the same dendrites each week. We placed a cranial window 3 weeks prior to 2-photon transcranial imaging onset and imaged weekly for up to 11 weeks (Fig. 2A). Data collection was ended (or weeks were excluded) when imaging quality was not sufficient to assure quantification of smaller spines, as determined by blinded investigators. Mice (age at imaging start, Control: 38.9 ± 5.3 weeks, *n* = 6; α-Syn OE: 39.9 ± 4.4 weeks, *n* = 8, mean ± SEM) were initially imaged for 2 weeks (Week − 1 and Week 0) to establish the pool of examinable dendrites and then injected with the relevant viruses (Fig. 2A, Fig. 1A). We then continued imaging the same dendrites for a further 9 weeks (Fig. 2A).

We found that spine density in mice overexpressing human α-Syn was lower than control animals in the week immediately following injection, but then significantly increased in the apical tufts of Layer V PFC neurons on a weekly basis, while spine density significantly decreased on a weekly basis in dendrites from mice injected with control virus (Fig. 2B, C; Control vs. α-Syn p = 0.006, week*treatment *p* < 0.001, linear mixed model). α-Syn OE mice had significantly higher spine density at week 5, 6, 8, and 9 following α-Syn OE, proportional to Week 0, via weekly Control vs. α-Syn OE comparisons. We also noted an increase in control dendritic spines 1-week post-injection, potentially in response to the viral transduction. However, this effect was no longer evident by Week 2 (Fig. 2C).

### Alpha-Synuclein Overexpression in the PFC Leads to Increased Persistence and Survival of New Spines, with no Significant Change in Formation or Elimination

The increased spine density we observed began at 5 weeks following injection with AAV-α-Syn and continued for the duration of the study (Fig. 2C). We, therefore, asked whether this increased spine density was due to alterations in generation of new spines, changes in elimination, or differential spine persistence. First, we investigated spine formation and elimination densities. To compensate for the infrequency of these events, formation and elimination densities per week were grouped into early (week 1–3), mid (week 4–6), and late (week 7–9). This analysis revealed no significant differences in Spine Formation (Fig. 3A) Mixed-effects repeated measures: TIME: *p* = 0.0987 F (1.991, 336.4) = 2.334, TREAT: (*p* = 0.8963, F (1, 230) = 0.01703) or spine elimination (Fig. 3B) TIME: *p* = 0.7919, F (1.981, 334.8) = 0.2309, TREAT: 0.5903, F (1, 230) = 0.2906. Next, we sought to determine if the increased density was, therefore, due to increased persistence of newly formed spines. Newly formed spines can be classified by whether they remain the week after they form (persistent) or recede (transient), so we identified what percentage of newly formed spines were still there the week after appearing (new persistent) and whether there were any differences in persistence between control and α-Syn OE groups following injection (Fig. 3C). It was not possible to identify persistence rates prior to injection given the limited number of imaging weeks.

This analysis demonstrated that dendrites from mice with local α-Syn OE had a significantly higher percentage of new-persistent spines in the weeks after injection (Odds ratios (OR) for spine survival by TREATMENT (syn vs. control): OR 1.30 CI 1.01–1.67, *p* = 0.040, and WEEK: 0.98, CI 0.94–1.02, *p* = 0.3). An interaction between time and TREATMENT was examined and not found to be significant (Fig. 3C). To investigate the survival of newly formed spines, we generated survival curves for each new spine identified during the first 3 weeks after the injection with AAV-mCherry and AAV-α-Syn. We found that dendritic spines in the α-Syn OE group survived for a significantly longer time that controls (Kaplan–Meier, *p* = 0.023, Fig. 3D). Overall, these data suggest a mechanism whereby an increase in new-spine survival and persistence leads to a relative increase in spine density by 5 weeks after α-Syn OE in the PFC.

### AAV-mCherry Inhibits Genetically Expressed YFP Fluorescence

While characterizing dendrites in vivo to determine whether they contained mCherry expression (using laser excitation at 1040 nm), we noted a potential confound. We found that dendrites that developed high levels of mCherry lost visualization or expression of YFP (Supplemental Fig. 2). Dendrites that demonstrated this loss of YFP over time were excluded from all analysis. Thus, in vivo experiments described above are limited to dendrites within 1 mm of the injection site (affected by the local microenvironment) but excludes dendrites with the highest potential transduction. The few dendrites that were positive for mCherry in vivo, but which did not show YFP loss were graphed separately for illustration (Supplemental Fig. 1). The finding that genetically encoded Thy-YFP is suppressed in cells with high levels of mCherry is important for design of future studies. We have not noticed this phenomenon with expression of α-Syn alone (not shown), and there is very little interference when mCherry expression is reduced using a preceding internal ribosome entry site (IRES, see confocal study below), but this lower expression level cannot be visualized well by 2-photon. Importantly for evaluating conclusions of this study, mCherry was used in both the treatment group (AAV-α-Syn mixed with AAV-mCherry) and controls (mCherry-only AAV). mCherry-positive dendrites and spines did not display overt evidence of degeneration; however, we did not attempt quantification of spine density using the red channel, as smaller spines could not be accurately compared once the YFP faded. To more accurately address the effect of local α-Syn, we also qualitatively scored the relative amount of transduction (passing axons and dendrites) at each micro-imaging location as an indicator of the relative exposure to localized expressed protein (Supplemental Fig. 1 C + D). These graphs are included in supplemental for illustrative purposes (given the low sample size in some sub-groups).

### Increased Dendritic Spine Density after Local α-Syn Overexpression is Primarily Driven by Cells Negative for Phosphorylated α-Syn

**Fig. 4:**
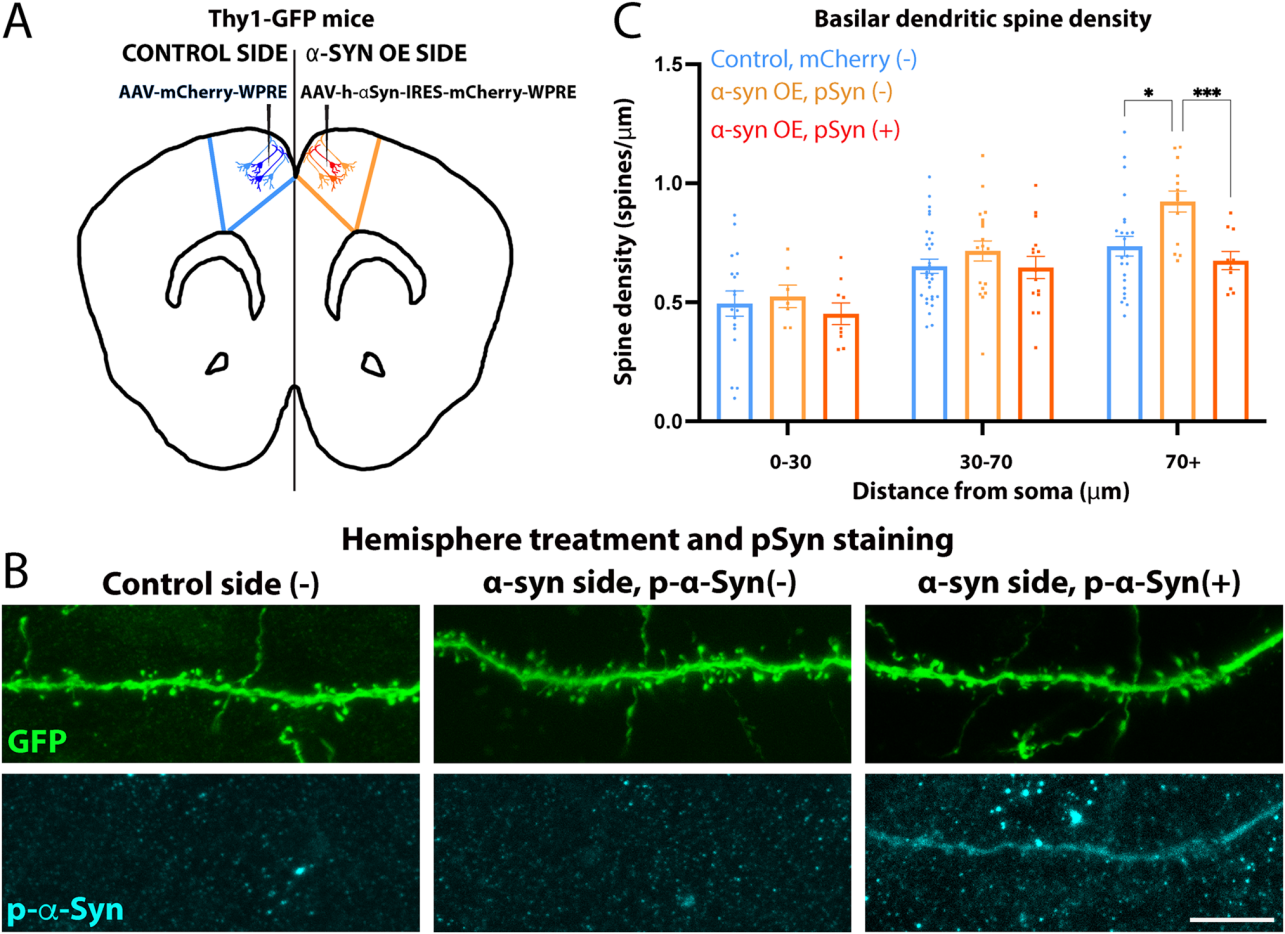
Increased dendritic spine density in response to alpha-Synuclein overexpression occurs in pSer129-negative neurons within the injection location. **A** Mice expressing Green fluorescent protein (GFP) in Layer V neurons (Thy1-GFP) mice were injected with control vector (AAV-mCherry) in one hemisphere and vector for α-Syn overexpression (OE): (AAV-α-Syn-IRES-mCherry) in the contralateral hemisphere for within animal comparison. **B** Spines from basilar dendrites 6 weeks post-injection were quantified via confocal microscopy. Sections were immunostained with antibodies against GFP and pSer129-α-Syn and dendritic spines were counted. **C** Basilar dendrites were analyzed in bins based on distance from the soma and grouped according to the hemisphere treatment (control or α-Syn OE). For the α-Syn OE hemisphere, cells were further divided into pSer129-α-Syn positive or negative. pSer129-α-Syn-negative dendrites had greater spine density than both mCherry-negative dendrites on the control hemisphere and pSer129-α-Syn-positive dendrites from the same hemisphere (Mixed-effects model, Main effect: treatment: F(2,63) = 3.397, *p* = 0.0397). Individual comparisons were corrected via Tukey: *p = 0.0106, ***p = 0.0010

There was a small subset of dendrites in the 2-photon dataset that could positively be identified during in vivo imaging as being transduced via mCherry expression (Supplemental Fig. 1A + B). However, most of the dendrites measured were mCherry-negative by 2-photon imaging, thus, likely non-transduced or transduced with lower-copy levels (as live 2-photon imaging is not as sensitive as confocal microscopy on fixed sections). To assess direct vs. indirect effects of α-Syn OE on spine density, we designed an experiment to more accurately measure spine density based on cells that contain pSer129-α-Syn (aka p-α-Syn). Because the Thy1-YFP line contains high numbers of labeled neurons in older adult animals in Layer V of the cortex, we utilized the Thy1-GFP line (Feng et al. 2000), which contains sparse GFP labeling in layer V. In each mouse, we overexpressed control protein in one PFC hemisphere and α-Syn + control protein in the contralateral hemisphere (AAV6-CAG-mCherry vs. AAV6-CAG-hSNCA-IRES-mCherry-WPRE). We collected brains 6 weeks after injection and immunostained for GFP and pSer129-α-Syn to identify p-α-Syn-positive and negative cells from the α-Syn OE hemisphere and compare them with control cells from the contralateral hemisphere. As with Thy-YFP animals, we again found that GFP fluorescence was reduced, in this case specifically when mCherry alone was overexpressed without the suppressive effect of the IRES. This prevented accurate spine density of mCherry-positive cells on the mCherry side, even when cells were stained using antibodies to GFP. By contrast, GFP-signal drop did not occur to the same extent when mCherry was expressed using a virus that containing “α-Syn-IRES-mCherry” (injected on the α-Syn OE side). We were, therefore, able to assess dendritic spine density of basilar dendrites of layer V pyramidal neurons to compare 3 groups: (1) control hemisphere/mCherry-negative; (2) α-Syn OE hemisphere/p-α-Syn-positive; and (3) α-Syn OE hemisphere/p-α-Syn-negative (Fig. 4A). Spine density was quantified in basilar dendrites based on distance from the soma. A repeated measure mixed-effects model was used to evaluate spine density across the length of the dendrite, showing a main effect of distance from soma (*p* < 0.0001, *F* (1.995, 74.82) = 31.69) and group (*p* = 0.0397, *F* (2, 63) = 3.397), and no interaction (Fig. 4B, C). Planned comparisons (corrected by Tukey’s multiple comparisons test) showed that dendrites from p-α-Syn-negative neurons on the treatment side had higher density compared with equivalent dendrites on the control side (mCherry-negative) in bins that were > 70um from the soma (*p* = 0.0106). They also had greater density than neighboring dendrites from p-α-Syn-positive neurons (*p* = 0.0010). Notably, dendrites from p-α-Syn-positive neurons did not show a significant difference in spine density compared with the control neurons, despite the presence of phosphorylated α-Syn in the cell body, dendrites, or dendritic spines (Fig. 4B). Our findings reveal that increased spine density early after localized α-Syn OE in the mouse PFC is likely driven by neighboring pSer129-α-Syn-negative neurons, while those expressing pSer129-α-Syn show no clear differences in spine density (Fig. 4B, C), in line with the overall results from our 2-photon experiments.

## Discussion

α-Syn pathology in the cortex is a defining pathological feature of dementia in synucleinopathies, yet it is unclear how pathology in the cortex impacts this region. Since α-Syn is a synaptic protein, we hypothesized local overexpression would alter dynamics of the synapse and local circuits, leading to alterations in dendritic spines. Turnover of dendritic spines in Layer V cortical pyramidal neurons is an essential functional process, allowing plasticity of these neurons within relevant circuitry (Gemin et al. 2021; Rajkovich et al. 2017). To isolate the effect of α-Syn overexpression on cortical cell function, we combined cranial windows with awake, longitudinal 2-photon imaging to track dendritic spine density before and 9 weeks following human-α-Syn OE in the mouse PFC. Our results suggest that early α-Syn OE targeted locally to the cortex leads to dynamic increases in spine density driven by increased survival of new-persistent spines, rather than significant changes in spine formation or elimination. To understand these results, we then used post-mortem confocal microscopy to compare spine density based on whether neurons showed phosphorylation of α-Syn, a pathological hallmark of the disease. Intriguingly, we found higher spine density within the injection site may be primarily driven by dendrites from neurons without α-Syn phosphorylation, rather than their neighboring neurons showing high p-α-Syn levels. Importantly, these neurons are surrounded by substantial numbers of presynaptic contacts expressing high levels of α-Syn (Fig. 1B). One possibility is that increased α-Syn in presynaptic partners may alter neurotransmitter release and induce a stabilizing effect. Interestingly, we did not detect loss of spines in p-α-Syn-positive neurons within the injection site compared with neurons on the control side, suggesting that this marker of pathological phosphorylation alone is not sufficient to cause spine loss. These results have implications for understanding the health of neurons within the local circuit where α-Syn expression is altered. Overall, these findings are consistent with our previous study in post-mortem mice and provide new insights into the etiology of this early response to overexpression.

There is some evidence that changes at the synapse precede neuronal cell loss that is characteristic of PD and LBD (Calo et al. 2016; Tanji et al. 2010; Kramer and Schulz-Schaeffer 2007); therefore, understanding early synaptic changes will likely prove beneficial to understanding disease progression as well as the role of native α-Syn. Some previous work has investigated the impact of α-Syn perturbations on synapses in vivo. For example, survival of dendritic spines in adult-born olfactory granule cells is decreased following overexpression of an aggregate-prone α-Syn mutant (Neuner et al. 2014). Similarly, turnover rates were higher in the somatosensory cortex of aged mice genetically overexpressing α-Syn, suggesting increased instability (Blumenstock et al. 2017). However, these studies have focused on transgenic models where differential influence of local pathology vs. impacts from changes in neuromodulators is not readily disambiguated. Independent analysis of individual regions allows for isolated modeling of aspects of PD/LBD biology to explore differential cell vulnerability, as heterogeneity in pathological distribution and symptoms are a key feature of synucleinopathies (Carceles-Cordon et al. 2023). Our results suggest that our early local cortical overexpression model displays some hallmarks of synuclein pathology (e.g., phosphorylation at Ser129, including at pre and post-synapses (Colom-Cadena et al. 2017)) and not others (staining is homogeneous, rather than clearly aggregated, Fig. 1F). Therefore, our results are most informative for understanding early responses to local upregulation of α-Syn.

Our previous study suggested that local increases of α-Syn in the PFC led to higher dendritic spine density at 10 weeks following viral overexpression using Golgi staining (Wagner et al. 2020). However, it was limited by a single timepoint. The results of our current work support the conclusions of our previous study and build on them by establishing a time-course, which shows that spine density changes can occur much sooner in response to α-Syn OE than previously recognized: at ~ 5 weeks post-injection. Previous work from other groups have also demonstrated that there are conditions which cause increased spine density in response to α-Syn OE. One study investigated human α-Syn OE in newly born dentate gyrus cells of the hippocampus under both the conditions of a transgenic mouse line (PDGF-h-α-Syn) or viral transduction of an overexpression construct (Winner et al. 2012). In the transgenic line, newly born neurons that overexpressed α-Syn within surrounding α-Syn OE tissue contained fewer dendritic branches, but higher spine density compared with newly born neurons in the non-transgenic line. Interestingly, individual neuronal transduction of α-Syn OE within wild-type tissue led to reduced dendritic spine density (Winner et al. 2012). Although our current study did not investigate dendrite arborization, these published data suggest that both the local microenvironment and influence of neighboring neurons, as well as α-Syn expression levels within neurons can influence spine density, highlighting autonomous vs. non-cell autonomous differences (Winner et al. 2012).

Our confocal analysis provides context regarding the microenvironment. Cells in the region positive for pSer129-α-Syn did not show changes in dendritic spine density compared with those on the contralateral hemisphere. By contrast, neurons within the transduced region but negative for pS129-α-Syn demonstrated a relative increase compared with neighboring and contralateral cells. One limitation was that while we were able to stain pSer129-α-Syn within individual neurons used for spine quantification, it was not possible to stain total (non-phosphorylated) synuclein at cellular resolution within the injected region (despite good staining elsewhere); we suspect that high α-Syn OE within the localized region prevents full penetration of the antibody through the tissue. Thus, it is possible that neurons with increased density have mildly elevated α-Syn levels that are below the level necessary to provoke pathological phosphorylation. Despite this limitation, our experiment demonstrates that the localized, regional overexpression of α-Syn induces increased spine density in cells without phosphorylated α-Syn, while cells with phosphorylated α-Syn show no difference from control cells at this time point. Given the large number of α-syn-positive axons in the region of the transduction site, it is also likely that presynaptic terminals from pSer129-α-syn-positive neurons interact with spines from the pSer129-α-syn-negative cells. One potential explanation for our results is that presynaptic α-Syn may have a stabilization effect on new spines, thereby increasing spine density. Interestingly and in contrast to previous studies, we did not detect any decreases in spine density in the dendrites that were pSer129-α-Syn positive. Whether this is a feature of differential vulnerability of cortical neurons compared to other cell types, or due to the overexpression time (6 weeks) used in the confocal study, will be intriguing to investigate in future studies.

Our findings are contrasted by multiple studies in the literature showing decreased spine density following α-Syn perturbations. For example, studies in the olfactory bulb (Neuner et al. 2014) and cortex (Blumenstock et al. 2017) of transgenic synuclein overexpression mice demonstrated reduced dendritic spine density. PDGF-h-α-Syn (α-Syn OE) mice have reduced cortical dendritic spine density starting at 3 months of age and persisting from 6 months onwards, along with greater turnover of spines in the OE group (Blumenstock et al. 2017). Interestingly, the decline in spine density did not get progressively worse after 6 months of age and no differences were seen between control and α-Syn OE mice in an earlier timepoint of 2 months (Blumenstock et al. 2017). Two major possibilities exist for the differences between our study and other models; (1) local overexpression presents neurons with different conditions within the context of an otherwise wild-type set of afferents from more distant brain regions. In other words, spine loss in other models may be due to dopaminergic or cholinergic depletion. (2) Our early timepoint represents a pre-symptomatic stage of α-Syn pathology where spine density is initially elevated. Future work to extend the experimental timeframe of imaging will be useful to delineate any potential ‘crossover’ point between increased spine density early and decreased density in response to chronic α-Syn OE.

The consequences of increased spine survival from our manipulation are not yet known. Experiences alter cortical dendritic spine anatomy and density (Nava et al. 2017; Pan et al. 2010); therefore, increased spine density can be advantageous under certain conditions. For instance, learning a new motor task leads to clustering of new spines near established spines that are related to a given task, and these spines are strengthened as the task familiarity increases in the primary motor (M1) cortex (Hedrick et al. 2022). By contrast, aberrant pruning and excessive dendritic spines are features of the loss of the protein associated with Fragile X Syndrome, which causes intellectual disability and autism (Grossman et al. 2010; Pan et al. 2010). Whether the increased spine density that we observe in response to α-Syn OE translates to measurable effects on PFC-mediated behavioral performance remains to be tested. Davidson et al. (2020) found that spine density naturally increases with age in mouse Layer V neurons of M1 cortex (Davidson et al. 2020). In some studies, hyperexcitability coincides with increased spine density (Chen et al. 2012) and may be detrimental long-term to neuronal circuitry. Intra-parenchymal AAV injection driving human α-Syn into rat neonates caused robust cortical overexpression (among other brain regions) and resulted in increased open field activity at 6 months of age, impaired neuronal health, hallmarks of apoptosis, and reduced cortical ChAT immunopositivity (Aldrin-Kirk et al. 2014).

The timeframe over which we measured the effects of α-Syn OE provides important insight into early responses to overexpression. Although these findings are relevant to individuals with genetic overexpression of α-Syn, the time course of the response of cells and circuits to this manipulation is also relevant for other pathologic and physiologic processes where alterations in expression or accumulation of this protein have been reported, such as autism, lead exposure or inflammation (Karaca et al. 2023; Zhang et al. 2012; Kasen et al. 2022). Advances in extended longitudinal tracking using prisms will allow future studies to determine if these changes persist or evolve over the lifetime of the animal and how they relate to behavioral consequences longitudinally.

### Limitations

One limitation of our study is that spines less than 0.45 µm (length) were not counted as they could not be distinguished from noise in vivo. Similarly, we found that classifications of spine morphology, which rely on visualizing the spine neck, were unreliable between blinded observers in the in vivo dataset and were, therefore, excluded from analysis. Thus, an alternative interpretation for the findings is that α-Syn increased the relative size, rather than number, of dendritic spines, leading to greater detection. This limitation is mitigated in part by our confocal study that allowed improved detection of smaller spines. Within the confocal study, spine density remained higher, and pSer129-α-syn-negative neurons on the AAV-α-Syn injected side were on average longer, but not wider than spines from the contralateral side (Supplemental Fig. 3).

Second, individual dendrites within the in vivo study had missing timepoints due to a variety of factors, including loss of window transparency and covid-19 pandemic related disruptions. For this reason, the primary outcomes were analyzed by independent statisticians to determine whether the conclusions were robust.

Finally, a major highlight of our study, distinct from prior experiments, was imaging of the same dendrites both before and after local viral overexpression, a powerful but challenging technique that allows evaluation of individual dendrites’ response to manipulation. However, this technique introduced two confounds described in the manuscript. First, introduction of the glass pipet directly at the site of imaging occasionally caused disruption or surface bleeding that may have impacted neuron health, spine density, and caused increased likelihood of premature opacity of the window. This limitation is likely to be equal between groups. Future use of a conditional, drug-driven strategy could mitigate this issue, but also has its own caveats as the drug could alter dendritic spines. Second, as discussed extensively above, high levels of mCherry expression driven by AAV (tested with two different promoters) reduced visualization of genetically encoded, Thy1-driven YFP and GFP. This finding significantly limited our ability to separately evaluate transduced and non-transduced neurons. For this reason, we focused on mCherry-negative cells exposed to the local microenvironment to avoid this confound. Although we did not include dendrites that showed disappearance of YFP over the course of the study in our analysis, the exclusion of these dendrites may have induced selection bias. Despite these limitations, this study shows a consistent response to early, local cortical overexpression and provides evidence that these changes are secondary to changes in spine survival.

## Conclusions

Lewy body diseases arise due to a complex interplay of genetic and protein perturbations that affect neuronal circuitry, which are then compounded by the long timeframe from prodromal to symptom onset. Evaluating the role of cortical α-Syn allows a better understanding of this complexity in this multi-brain-region, multi-system disease. The long-term goal is to understand the relative balance and interconnection of pathology in diverse brain regions, encompassing the heterogeneity from Parkinson’s disease with motor symptoms to pure-psychiatric onset disease. These insights are key to devising effective therapies to treat diverse Lewy-body-related symptoms, as well as to understanding other physiologic and pathologic conditions that alter α-Syn levels.

## Supplementary Information

Below is the link to the electronic supplementary material.

Supplementary file1 (DOCX 27663 KB)

## Data Availability

No datasets were generated or analysed during the current study.
