# Supplementary material for: Enhanced Spine Stability and Survival Lead to Increases in Dendritic Spine Density as an Early Response to Local Alpha-Synuclein Overexpression in Mouse Prefrontal Cortex
Source: Cell Mol Neurobiol. 2024 Apr 26;44:42. doi: 10.1007/s10571-024-01472-7 (PMC11052719; doi:10.1007/s10571-024-01472-7)
Supplement: Supplementary file 1 — Supplementary file1 (DOCX 27663 KB) [file 10571_2024_1472_MOESM1_ESM.docx]

**
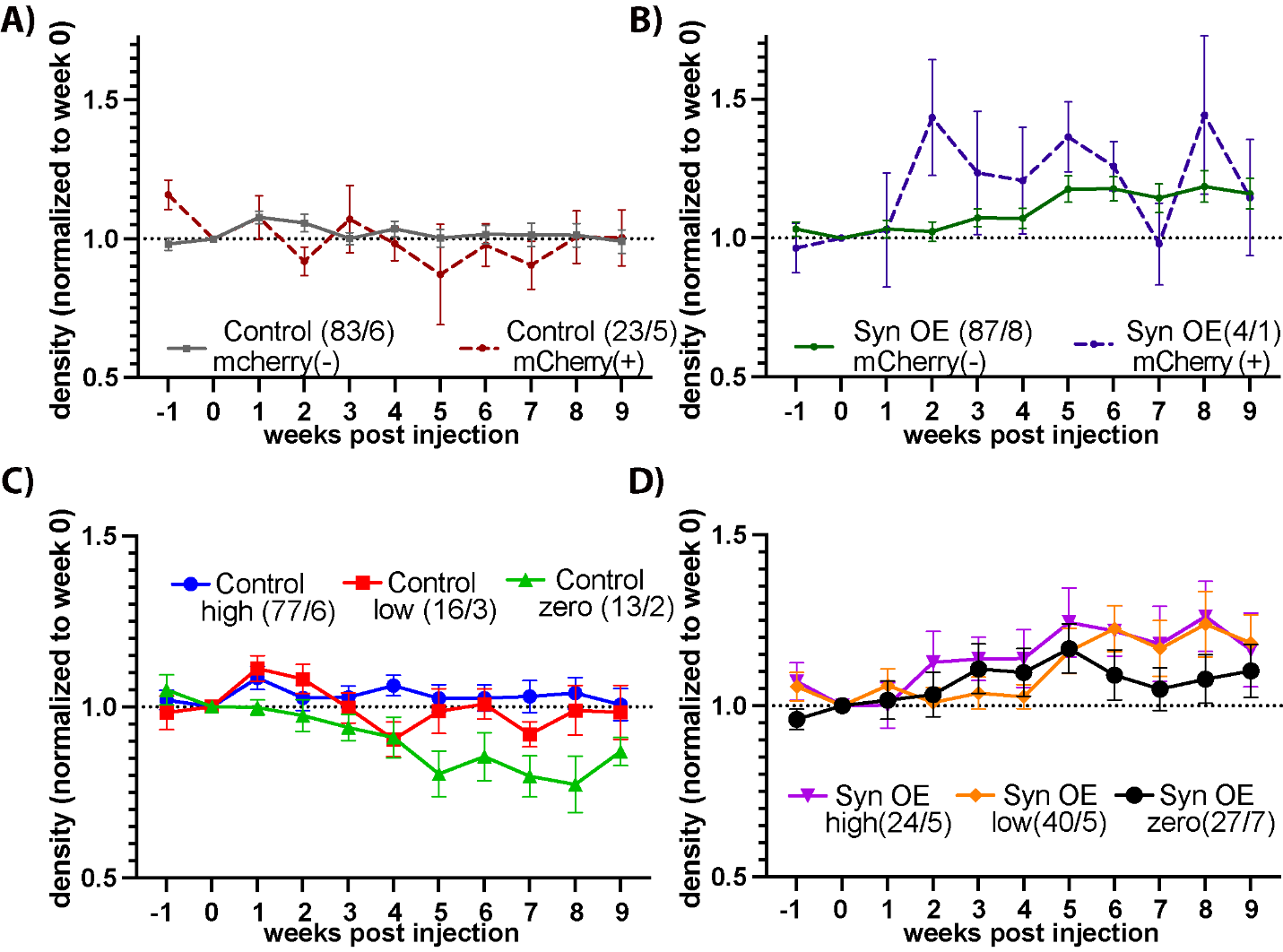
**

**Supplemental Figure 1: Effect of Transduction (A, B):** Relative change in spine density based on detection (-/+) of mCherry at 1040 nm during live imaging, in mice injected with AAV- mCherry (aka. Control, A), or α-Syn overexpression (Syn OE: mixed AAV-mCherry + AAV-α-Syn, B). Few mCherry positive dendrites could be reliably counted because high levels of mCherry caused loss of YFP, and low levels could not be detected by 2-photon at 1040nm. Dendrites with ambiguous transduction at 1040nm were excluded from this graph. Total identified dendrites from # of mice are listed in parathesis (dendrites/mice) but were not available for all weeks. **Local Microenvironment (C, D)**: As α-Syn may be secreted or impact presynaptic partners, we graphed how amount of regional transduction in the layer I microenvironment affected dendrites localized to this area in mCherry injected mice (C) and in SynOE mice (D). Sub-analyses for illustrative purposes only; no statistics were performed given the small sample size within groups/weeks.

**
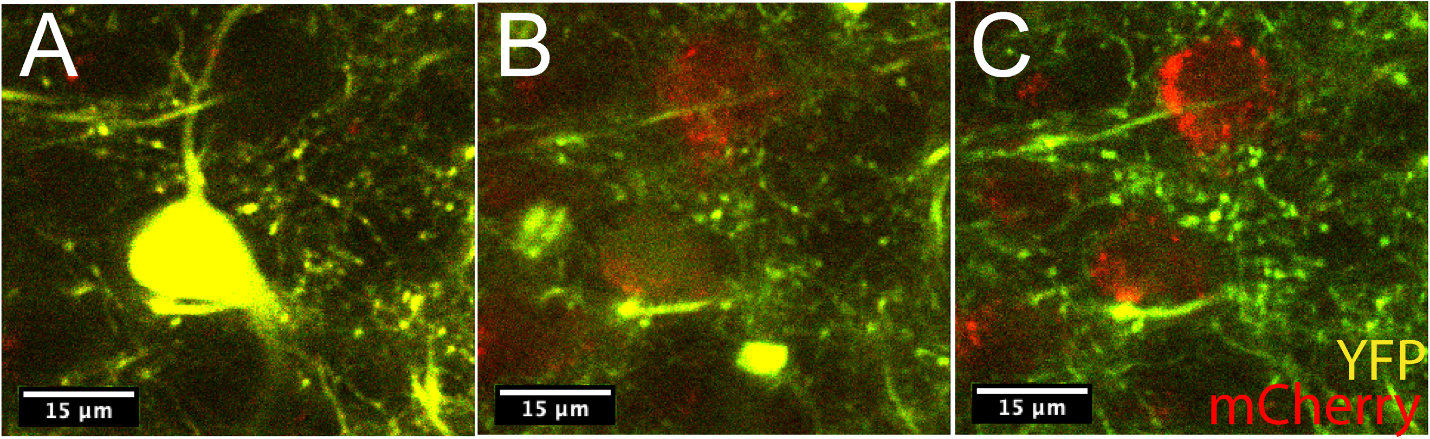
**

**Supplemental Figure 2:** Detection of Yellow-fluorescent protein (YFP) expression in the Thy-1 YFP-H mouse line decreases following transduction with AAV6-mCherry. (A) pre-injection cell body, (B) 8 weeks following injection the YFP is barely visible in the cell body and mCherry is present, and (C) 11 weeks following injection YFP is no longer detectable in this cell and mCherry is clear. (Displayed image at 935nm, which favors YFP). Similar issues were found with different transgenic models (Thy1-GFP-M line), different serotypes (AAV1) and different promoters (hSYN vs. CAG). This phenomenon is mitigated when mCherry is preceded by IRES to reduce expression, as in our confocal experiment (See text), but this lower expression level is difficult to visualize in vivo.


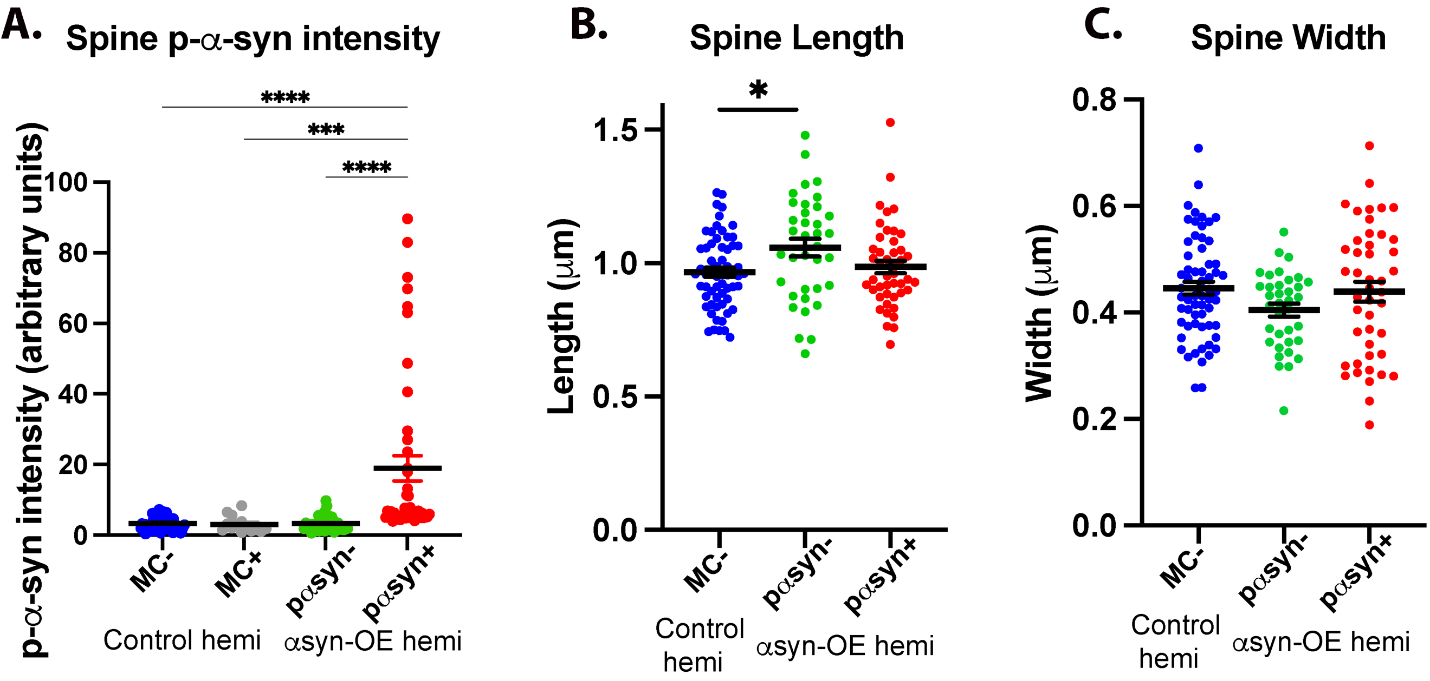


**Supplemental Figure 3:** A) There is overall higher average p-α-Syn levels in spines from p-α-Syn+ neurons in the α-Syn overexpression hemisphere (α-Syn-OE hemi), compared with mCherry negative (MC-) and positive (MC+) neurons in the control hemisphere. B) Spine length is longer in p-α-Syn negative neurons in the α-Syn-OE-hemisphere compared with MC- neurons in the control hemisphere. C) No significant differences were detected in spine width. MC+ neurons in the control hemisphere were not analyzed due to reduced GFP (see limitations and supplemental figure 2 for details).*p < 0.05, **p <0.01, ***p <0.001, ****p<0.0001, via ANOVA followed by Tukey’s multiple comparison’s test. Confocal analysis from N=4 Thy1-GFP mice.
